# Sociodemographic Inequalities in Health Insurance Ownership among Women in Selected Francophone Countries in Sub-Saharan Africa

**DOI:** 10.1155/2021/6516202

**Published:** 2021-08-17

**Authors:** Yiting Wang, Xuhui Wang, Lu Ji, Rui Huang

**Affiliations:** ^1^School of Business, Central South University, Changsha 410083, China; ^2^Hunan University of Finance and Economics, Changsha 410205, China; ^3^Guangdong Women and Children Hospital, China; ^4^School of Medicine and Health Management, Tongji Medical College, Huazhong University of Science and Technology, China; ^5^School of Pharmacy, Tongji Medical College, Huazhong University of Science and Technology, Wuhan, China

## Abstract

In sub-Saharan Africa, improving equitable access to healthcare remains a major challenge for public health systems. Health policymakers encourage the adoption of health insurance schemes to promote universal healthcare. Nonetheless, progress towards this goal remains suboptimal due to inequalities health insurance ownership especially among women. In this study, we aimed to explore the sociodemographic factors contributing to health insurance ownership among women in selected francophone countries in sub-Saharan Africa. *Methods*. This study is based on cross-sectional data obtained from Demographic and Health Surveys on five countries including Benin (*n* = 13,407), Madagascar (*n* = 12,448), Mali (*n* = 10,326), Niger (*n* = 12,558), and Togo (*n* = 6,979). The explanatory factors included participant age, marital status, type of residency, education, household wealth quantile, employment stats, and access to electronic media. Associations between health insurance ownership and the explanatory factors were analyzed using multivariate regression analysis, and effect sizes were reported in terms in average marginal effects (AMEs). *Results*. The highest percentage of insurance ownership was observed for Togo (3.31%), followed by Madagascar (2.23%) and Mali (2.2%). After stratifying by place of residency, the percentages were found to be significantly lower in the rural areas for all countries, with the most noticeable difference observed for Niger (7.73% in urban vs. 0.54% in rural women). Higher levels of education and wealth quantile were positively associated with insurance ownership in all five countries. In the pooled sample, women in the higher education category had higher likelihood of having an insurance: Benin (AME = 1.18; 95% CI = 1.10, 1.27), Madagascar (AME = 1.10; 95% CI = 1.05, 1.15), Mali (AME = 1.14; 95% CI = 1.04, 1.24), Niger (AME = 1.13; 95% CI = 1.07, 1.21), and Togo (AME = 1.17; 95% CI = 1.09, 1.26). Regarding wealth status, women from the households in the highest wealth quantile had 4% higher likelihood of having insurance in Benin and Mali and 6% higher likelihood in Madagascar and Togo. *Conclusions*. Percentage of women who reported having health insurance was noticeably low in all five countries. As indicated by the multivariate analyses, the actual situation is likely to be even worse due to significant socioeconomic inequalities in the distribution of women having an insurance plan. Increasing women's access to healthcare is an urgent priority for population health promotion in these countries, and therefore, addressing the entrenched sociodemographic disparities should be given urgent policy attention in an effort to strengthen universal healthcare-related goals.

## 1. Introductions

Women's access to healthcare is regarded as an important indicator of the quality and performance of healthcare systems [1–3]. Ensuring sustainable access to adequate and equitable care plays an instrumental role in preventing maternal and child mortality and associated adverse outcomes at social and healthcare levels. With the growing understanding of the repercussions of inequality in healthcare caused by various community and healthcare level barriers, the importance of promoting universal healthcare and its facilitators such as health insurance is becoming increasingly apparent [4–6]. Owing to diverse issues such as lower socioeconomic status and inadequate decision making power, women in many sub-Saharan countries are more likely have unmet needs of healthcare and face disproportionately higher burdens of preventable morbidity and mortality arising from obstetric complications [7, 8]. During their lifetime, women require more frequent medical contacts than men and thus incur higher healthcare-related costs which place them at a greater vulnerability to poorer health, impoverishment, and socioeconomic marginalization. Healthcare financing strategies to reduce financial barriers to medical care among women will not only contribute to better maternal and child health but also to women's empowerment opportunities such as education and labour market participation which in turn can improve health outcomes and spur economic growth of countries. While achieving universal health insurance coverage is far from being a reality in African countries, health policymakers must strive to address the socioeconomic inequalities in health insurance subscription and catastrophic health expenditures among vulnerable population groups and among women of reproductive age in particular [9, 10].

World Health Organization also recognizes the challenges to meet the healthcare needs of women with scarce resources for low-middle-income-countries in Asia and sub-Saharan Africa. Achieving universal health coverage will require continuous effort and innovative planning and making the best use of the available resource and evidence. Experts around the world suggest that health insurance is one of the most promising tools for achieving universal health coverage and protecting the health of the disadvantaged population [11, 12]. A growing body of literature provides evidence on the ongoing discussion regarding the importance and mechanisms of insurance reforms and healthcare financing techniques to cover the healthcare needs of the uninsured population. In a report titled “Why Health Insurance Matters,” the Institute of Medicine (US) Committee on the Consequences of Uninsurance maintained that health insurance pools the risks and resources of a large group of people so that each is protected from financially disruptive medical expenses resulting from an illness, accident, or disability [13]. Increasing the number of people covered by health insurance plans constitutes a key strategy to achieving universal healthcare and thereby meeting the Sustainable Development Goal (SDG 3.8) of safeguarding the vulnerable population from financial risk resulting from catastrophic health expenditures [14]. In sub-Saharan Africa, the barriers to accessing medical services are challenging among women due to the inequalities in social determinants such as inadequate opportunities for socioeconomic empowerment and meeting their special healthcare needs. The challenges for achieving universal healthcare coverage (UHC) in francophone countries were reported previously. In the present study, we aimed to explore the sociodemographic divide in health insurance ownership among women in selected francophone countries in sub-Saharan Africa [15]. Several studies have attempted to explore the factors associated with health insurance ownership [16–19], but they are mostly based on small scale samples, and findings are not comparable across the studies due to methodological and measurement heterogeneity. This study addresses this gap by analyzing data that are structurally uniform, and the sample population are nationally representative as well.

## 2. Methods

The present study was based on open-access data collected from Demographic and Health Surveys (DHS) in the following countries: Benin (survey year = 2017-18; *n* = 15,928; response rate = 98%), Madagascar (survey year = 2008-09; *n* = 17,375; response rate = 96%), Mali (survey year = 2012; *n* = 10,424; response rate = 95.9%), Niger (survey year = 2012; *n* = 11,160; response rate = 95%), and Togo (survey year = 2013; *n* = 9,840; response rate = 98%). All of these surveys are nationally representative and cover community sample population, e.g., residing in households. For this study, we obtained data on adult women aged 15-49 years. DHS surveys are conducted by joint collaboration by U.S. Agency for International Development (USAID), the United Nations Children's Fund (UNICEF), and the United Nations Population Fund (UNFPA) with technical assistance for the survey was provided by ICF international. The main objectives of the survey were to collect data on key demographic indicators such as fertility, childhood mortality, and maternal and child health status. Data serve the purpose of measuring the progress towards national and international development goals (such as Sustainable Development Goals) and facilitating evidence-based policies.

### 2.1. Variables

The outcome variable was insurance ownership. This was measured by asking the main respondent about insurance ownership of household members. Answer to this question was categorised as “Covered by health insurance” and “Not covered.” Several enabling and predisposing factors were chosen as the predictor variables based on their theoretical association with insurance ownership that is described in Table 1. We also conducted a literature search to identify the potential predictor variables of insurance ownership.

### 2.2. Data Analysis

Data were analyzed with Stata version 16 (College Station, TX: Stata Corp LP). All analyses were adjusted for the cluster design by using the svy command. This command uses the information on sampling weight, strata, and primary sampling unit provided with the datasets.  Table 2 is described as percentages with 95% confidence intervals. Percentages of respondents who reported having any health insurance were described separately for each country. Following that, binary logistic regression models were used to estimate the associations between health insurance ownership and the explanatory factors. Using the “margins” functionality of Stata, these results were reported in terms of average marginal effects with 95% confidence intervals. At first, we ran a pooled model including all the countries, which was followed by a country-stratified analysis. Variance inflation factor (VIF) command was used to test for multicollinearity. No multicollinearity was detected as VIF values were below 10 for all the models. All tests were two-tailed and were considered significant at an alpha value of 5%. Model performance was assessed using receiver operating characteristic curve (ROC curve). The final step of the analysis involved calculation of percentage contribution of the variables to the total variance in the outcome factor for each of the five countries to highlight cross-country differences in the relative importance of the explanatory variables.

## 3. Results

**Figure 1:**
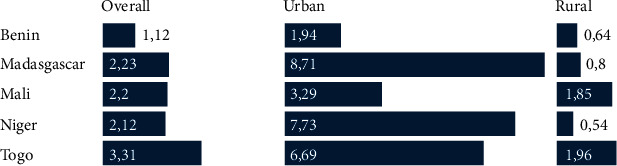
Percentage of participants with a health insurance by country.

As indicated by Figure 1, the highest percentage of insurance ownership was observed for Togo (3.31%), followed by Madagascar (2.23%) and Mali (2.2%). These cross-country differences in insurance ownership were (*p* < 0.001) statistically significant. After stratifying the percentage by place of residency, the percentages were found to be significantly lower in the rural areas for all countries, with the most noticeable difference observed for Niger (7.73% in urban vs. 0.54% in rural women). These regional differences were also statistically significant (*p* < 0.001).

Table 3 shows the percentage of health insurance ownership by sociodemographic characteristics such as age groups and place of residency. The results of multivariate logistic regression calculating the association between health insurance ownership with the sociodemographic factors are presented in Table 4. Age, marital status, and place of residency did not show any noticeable correlation with insurance ownership, whereas educational level and wealth status were positively associated with insurance ownership in all five countries. For instance, women in the higher education category had likelihood of having an insurance both in the pooled (AME = 1.14; 95% CI = 1.11, 1.17) and country specific analysis: Benin (AME = 1.18; 95% CI = 1.10, 1.27), Madagascar (AME = 1.10; 95% CI = 1.05, 1.15), Mali (AME = 1.14; 95% CI = 1.04, 1.24), Niger (AME = 1.13; 95% CI = 1.07, 1.21), and Togo (AME = 1.17; 95% CI = 1.09, 1.26). Positive effect on having a plan was observed for all higher quintiles of wealth as well. In the pooled sample, women from the households with higher and highest wealth quintile had 3% (AME = 1.03; 95% CI = 1.01, 1.07) and 4% (AME = 1.04; 95% CI = 1.03, 1.05) higher likelihood of having insurance, respectively. At country level, Benin and Mali had 4%, and Madagascar and Togo had 6% higher likelihood of having an insurance. In Madagascar, Niger, and Togo, having access to electronic media was associated with 3% higher likelihood of having an insurance.

**Figure 2:**
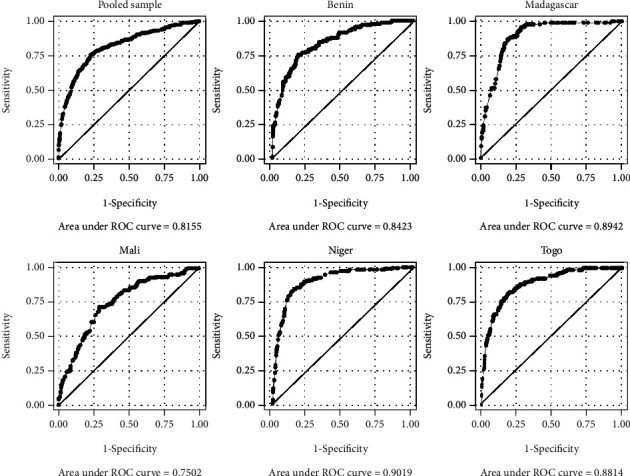
Receiver operating curves.

Following the regression analyses, we constructed receiver operating characteristic (ROC) curve to assess the predicting power of the models for the pooled sample and the individual countries as well. As shown in Figure 2, the ROC curve value was ranged from 0.75 to 0.90 which is considered good [21].

**Figure 3:**
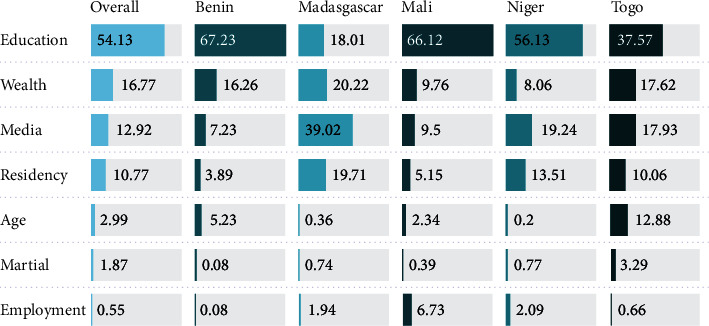
Relative importance of the variables in the equations.

Figure 3 shows the relative importance of the variable in explaining the total variance in insurance ownership in terms of percentage contributions. As indicated by the chart, access to media was the largest contributor to insurance ownership in the overall sample, as well as in DR Congo and Gabon. For Burkina Faso, education was the most important predictor (72.6% variance) compared with wealth status (36.9%) in Cameroon and age (33%) in Kenya.

## 4. Discussion

The aim of the present study was to report the prevalence of health insurance ownership and its sociodemographic correlates among adult women aged between 15 and 49 years in Benin, Madagascar, Mali, Niger, and Togo [22]. We used cross-sectional data from Demographic and Health Surveys conducted between 2012 and 2018. Population-based research on health insurance is important to measure progress toward Sustainable Development Goal 3.8 of safeguarding the vulnerable population from financial risk resulting from catastrophic health expenditures. Increasing the proportion of insured population and reducing the inequalities among socioeconomic groups is an important priority if universal healthcare insurance is to be achieved. Our findings from Demographic and Health Surveys revealed significant between and within-country disparities in insurance ownership among women. The highest percentage of insurance ownership was observed for Togo (3.31%), followed by Madagascar (2.23%) and Mali (2.2%) which are noticeably low in comparison with developed countries such as USA where 91.2% of the population had a health insurance as of 2017 [23]. The overall statistics are likely to be even more dismal since we included only women and the age group of 15-49 years.

Currently, there are no statistics on insurance ownership in these countries. A recent cross-sectional study conducted in Parakou, Benin, on 50 patients reported that the contribution of mutual health insurance to access to healthcare was marginal as most people could not afford this insurance [24]. Similarly, an earlier study published in 2004 reported that only 27% of the heads of household heads had permanent financial access to healthcare compared with 9% among the poorest [25], highlighting the greater need for health insurance interventions among the poor. In Madagascar, a pilot study reported that less than a third of people in need of healthcare accessed treatment when a payment of fees was involved compared with 65% when the fees were exempted, indicating the beneficial role of removing user-side fees on promoting healthcare access [26]. In Mali, membership with mutual health organizations (MHOs) was higher among the richest 20% of the households compared with the poorest 20% [27], another indication of the fact that financial barriers prevent the poor from accessing health insurance. Intuitively, for the poorest of the households, having to pay for premiums to have a plan that is of their financial reach can be as prohibitory as user fees to access medical care. Eliminating user fees is therefore critical to realize universal healthcare in sub-Saharan Africa [28].

Further analysis indicated significant urban-rural differences for all five countries such that participants in the rural areas had lower percentage of having a plan. Urban-rural differences in health insurance ownership were previously reported in China [29] and the USA [30] as well. Of note, the urban-rural gap was not significant in the multivariate regression analysis. Multivariate analysis indicated that insurance ownership was associated with women's educational level, household wealth status, and media access, reflecting a positive impact of women's socioeconomic status on insurance ownership [30, 31]. Findings indicated that women who had higher educational status and from higher wealth quantile households had higher likelihood of having health insurance. This can be explained in light of the arguments that socioeconomically empowered women are generally more likely to be aware of potential health-related expenditure and capable of affording insurance plans. It is therefore of utmost importance that socioeconomic disparities in insurance ownership be reduced among women to ensure equitable access to essential healthcare services, e.g., sexual and reproductive healthcare. The final step of the analysis involved calculating the relative importance of the predictor variables in the equation which captured the variation in the significance of the individual factors in insurance ownership in each country. Varying degrees (in terms of percentage contribution to the outcome factor) of contribution of the individual factors may imply that context-oriented and locally tailored interventions are necessary to improve insurance ownership.

This study makes a significant contribution to the literature on health insurance ownership among women in selected countries in sub-Saharan Africa. Data on demographics and other critical health indicators are extremely scarce for most countries in the African continent. From this perspective, Demographic and Health Surveys provide a crucial source to fill this data gap and facilitate evidence-based population health planning and intervention in the beneficiary countries. This has several strengths that are worthy of mentioning. The datasets contained large sample size and are nationally representative. Therefore, the findings are generalisable for the entire women population. In low-income settings, gathering data on large sample is constrained by budgetary limitations and lack of adequate research infrastructure. Furthermore, small-scale studies are generally conducted in line with the local circumstances and are usually not comparable to those from other settings due to variations in methodological approach and measurements of variables. From this viewpoint, our study has an additional advantage since measurements are homogenous in structure and measurements are also standardised to allow cross-cultural comparison. We have some important limitations to report as well. First, these are cross-sectional surveys, and therefore, our analyses cannot guarantee any causal relationship between the outcome and explanatory factors. Since the surveys are secondary, the choice of the variables was contingent on their availability in the datasets. There were also no details regarding the types of insurance schemes owned by the participants such as whether or not they are public or private, source of funding, and the types of services they covered. Further studies need to be conducted to address these limitations.

## 5. Conclusion

In this cross-sectional study, we reported the prevalence of health insurance ownership and its sociodemographic correlates among women of reproductive age in five francophone countries in sub-Saharan Africa including Benin, Madagascar, Mali, Niger, and Togo. The challenges for achieving universal healthcare coverage (UHC) in francophone countries were reported previously, and thus, our findings make an important contribution towards making evidence-based decisions in promoting health insurance ownership—a key instrument to achieving UHC—and among women of reproductive age—a key priority for global public health promotion. Our findings reflect a low level of health insurance ownership in all five countries, ranging from 1.12% in Benin to 3.31% in Togo. Multivariate analysis revealed significant sociodemographic disparities in insurance ownership especially in terms of educational status, household wealth index, and access to electronic media. Further analysis suggested important variations in the degrees to which these factors influence insurance ownership. These findings warrant the need for addressing the sociodemographic inequalities in health insurance ownership among women by taking into account the country-specific contextual factors.

## Figures and Tables

**Table 1:** Description of the variables included in the analysis.

Variables	Codebook	Description
Outcome variable		
Covered by health insurance	No (0), yes (1)	Whether or not a respondent is currently insured
Explanatory variables		
Age	15-19, 20-24, 25-29, 30-34, 35-39, 40-44, and 45-49 years	Age of respondent at the time of survey
Marital status	Not married (1), married (2)	Current marital status
Residency	Urban (1), rural (2)	Type of place residency
Education	None/less than primary (0), primary (1), secondary (2), higher (3)	Educational level based on total number of years of schooling
Wealth quintile	Poorest (1), poorer (2), middle (3), richer (4), richest (5)	Wealth index calculated based on possession of durable goods by a household [20]
Employment	No (0), yes (1)	Has outdoor employment
Media access	No (0), yes (1)	Has access to TV and radio

**Table 2:** Sample characteristics.

Variables	% (95% CI)
Age	
15-19	5.8 (5.6, 6.0)
20-24	20.4 (20.0, 20.7)
25-29	28.2 (27.8, 28.6)
30-34	22.3 (21.9, 22.7)
35-39	14.5 (14.1, 14.8)
40-44	6.7 (6.5, 6.9)
45-49	2.2 (2.1, 2.4)
Marital status	
Not married	15.0 (14.7, 15.3)
Married	85.0 (84.7, 85.3)
Residency	
Urban	26.9 (26.5, 27.3)
Rural	73.1 (72.7, 73.5)
Education	
No education	68.3 (67.9, 68.7)
Primary	20.1 (19.8, 20.5)
Secondary	10.8 (10.5, 11.0)
Higher	0.8 (0.7, 0.9)
Wealth quintile	
Poorest	22.9 (22.5, 23.2)
Poorer	20.2 (19.9, 20.6)
Middle	19.6 (19.2, 19.9)
Richer	18.5 (18.2, 18.9)
Richest	18.8 (18.5, 19.2)
Employment	
No	40.2 (39.8, 40.7)
Yes	59.8 (59.3, 60.2)
Media access	
No	63.9 (63.3, 64.5)
Yes	36.1 (35.5, 36.7)

**Table 3:** Health insurance ownership by sociodemographic characteristics (*n* = 49,493).

Variables	Total	No	Yes	*p*
Age				
15-19	5.8 (5.6, 6.0)	5.8 (5.6, 6.0)	4.0 (2.8, 5.3)	
20-24	20.4 (20.0, 20.7)	20.5 (20.1, 20.9)	13.5 (11.4, 15.6)	
25-29	28.2 (27.8, 28.6)	28.2 (27.8, 28.6)	27.1 (24.4, 29.9)	
30-34	22.3 (21.9, 22.7)	22.2 (21.8, 22.5)	27.6 (24.9, 30.4)	
35-39	14.5 (14.1, 14.8)	14.4 (14.1, 14.7)	17.2 (14.9, 19.5)	
40-44	6.7 (6.5, 6.9)	6.7 (6.5, 6.9)	8.6 (6.9, 10.3)	
45-49	2.2 (2.1, 2.4)	2.2 (2.1, 2.4)	1.9 (1.0, 2.7)	≤0.01
Marital status				
Not married	15.0 (14.7, 15.3)	15.1 (14.8, 15.4)	11.5 (9.5, 13.4)	
Married	85.0 (84.7, 85.3)	84.9 (84.6, 85.2)	88.5 (86.6, 90.5)	≤0.01
Residency				
Urban	26.9 (26.5, 27.3)	26.2 (25.8, 26.6)	61.5 (58.5, 64.5)	
Rural	73.1 (72.7, 73.5)	73.8 (73.4, 74.2)	38.5 (35.5, 41.5)	≤0.01
Education				
No education	68.3 (67.9, 68.7)	69.1 (68.7, 69.5)	30.7 (27.8, 33.5)	
Primary	20.1 (19.8, 20.5)	20.1 (19.8, 20.5)	20.1 (17.6, 22.6)	
Secondary	10.8 (10.5, 11.0)	10.2 (9.9, 10.5)	36.9 (34.0, 39.9)	
Higher	0.8 (0.7, 0.9)	0.6 (0.5, 0.6)	12.3 (10.3, 14.3)	≤0.01
Wealth quintile				
Poorest	22.9 (22.5, 23.2)	23.2 (22.9, 23.6)	5.8 (4.4, 7.3)	
Poorer	20.2 (19.9, 20.6)	20.5 (20.1, 20.8)	8.1 (6.4, 9.8)	
Middle	19.6 (19.2, 19.9)	19.8 (19.4, 20.1)	8.8 (7.0, 10.5)	
Richer	18.5 (18.2, 18.9)	18.6 (18.3, 19.0)	15.2 (13.0, 17.4)	
Richest	18.8 (18.5, 19.2)	17.9 (17.6, 18.3)	62.1 (59.1, 65.1)	≤0.01
Employment				
No	40.2 (39.8, 40.7)	40.4 (40.0, 40.8)	31.7 (28.8, 34.6)	
Yes	59.8 (59.3, 60.2)	59.6 (59.2, 60.0)	68.3 (65.4, 71.2)	≤0.01
Media access				
No	63.9 (63.3, 64.5)	65.1 (64.5, 65.6)	22.7 (19.7, 25.8)	
Yes	36.1 (35.5, 36.7)	34.9 (34.4, 35.5)	77.3 (74.2, 80.3)	≤0.01

N.B. For total sample column, percentage was reported with 95% CIs in parenthesis. For health insurance, row percentage was reported.

**Table 4:** Results of multivariate logistic regression estimations of the proportions of insurance ownership regressed on the sociodemographic factors in five SSA countries.

	Overall	Benin	Madagascar	Mali	Niger	Togo
Age (15-19)						
20-24	0.99^∗^ [0.98, 1.00]	0.99 [0.97, 1.01]	1.01 [0.98, 1.05]	0.98 [0.96, 1.00]	1.00 [0.98, 1.02]	1.00 [0.97, 1.03]
25-29	1.00 [0.99, 1.01]	0.99 [0.97, 1.01]	1.02 [0.99, 1.05]	0.99 [0.97, 1.01]	1.02^∗^ [1.00, 1.04]	1.01 [0.98, 1.03]
30-34	1.01 [1.00, 1.02]	1.00 [0.98, 1.02]	1.01 [0.98, 1.04]	1.01 [0.99, 1.04]	1.02 [1.00, 1.03]	1.03 [1.00, 1.05]
35-39	1.01 [1.00, 1.02]	1.00 [0.98, 1.02]	1.02 [0.98, 1.05]	0.99 [0.97, 1.01]	1.00 [0.98, 1.02]	1.05^∗∗∗^ [1.02, 1.09]
40-44	1.02^∗^ [1.00, 1.03]	1.01 [0.99, 1.04]	1.01 [0.97, 1.04]	1.01 [0.98, 1.04]	1.01 [0.99, 1.04]	1.07^∗∗^ [1.03, 1.12]
45-49	1.00 [0.98, 1.02]	0.99 [0.96, 1.01]	1.02 [0.94, 1.12]	0.99 [0.95, 1.02]	1.02 [0.98, 1.07]	1.04 [0.98, 1.09]
Marital status (single)						
Married	1.01^∗∗∗^ [1.01, 1.01]	1.00 [0.99, 1.01]	1.01 [0.99, 1.03]	1.00 [0.98, 1.02]	1.02^∗^ [1.00, 1.03]	1.01^∗^ [1.00, 1.03]
Residency (urban)						
Rural	1.00 [1.00, 1.01]	1.01 [1.00, 1.02]	0.99 [0.97, 1.01]	1.00 [0.99, 1.02]	0.99 [0.98, 1.01]	1.03 [1.00, 1.05]
Education (no education)						
Primary	1.01^∗∗^ [1.00, 1.01]	1.00 [0.99, 1.01]	1.04^∗∗^ [1.01, 1.06]	1.01 [1.00, 1.03]	1.01^∗^ [1.00, 1.02]	1.01 [1.00, 1.02]
Secondary	1.04^∗∗∗^ [1.03, 1.04]	1.02^∗∗∗^ [1.01, 1.03]	1.02^∗^ [1.00, 1.05]	1.07^∗∗∗^ [1.03, 1.10]	1.06^∗∗∗^ [1.04, 1.08]	1.04^∗∗∗^ [1.02, 1.06]
Higher	1.14^∗∗∗^ [1.11, 1.17]	1.18^∗∗∗^ [1.10, 1.27]	1.10^∗∗∗^ [1.05, 1.15]	1.14^∗∗^ [1.04, 1.24]	1.13^∗∗∗^ [1.07, 1.21]	1.17^∗∗∗^ [1.09, 1.26]
Wealth quintile (poorest)						
Poorer	1.01^∗∗∗^ [1.00, 1.01]	1.00 [0.99, 1.00]	1.00 [1.00, 1.01]	1.03^∗∗∗^ [1.02, 1.05]	1.01 [0.99, 1.02]	1.00 [0.99, 1.01]
Middle	1.00^∗^ [1.00, 1.01]	1.00 [1.00, 1.01]	1.00 [1.00, 1.01]	1.01^∗^ [1.00, 1.02]	1.00 [0.99, 1.01]	1.00 [0.99, 1.01]
Richer	1.03^∗∗∗^ [1.01, 1.07]	1.01^∗∗^ [1.00, 1.02]	1.02^∗^ [1.00, 1.04]	1.01^∗^ [1.00, 1.03]	1.01 [0.99, 1.02]	1.03^∗∗^ [1.01, 1.06]
Richest	1.04^∗∗∗^ [1.03, 1.05]	1.04^∗∗∗^ [1.02, 1.06]	1.06^∗∗∗^ [1.03, 1.09]	1.04^∗∗∗^ [1.02, 1.06]	1.03^∗∗∗^ [1.02, 1.05]	1.06^∗∗^ [1.02, 1.10]
Has employment (no)						
Yes	1.00 [1.00, 1.01]	1.00 [0.99, 1.01]	1.02^∗^ [1.00, 1.03]	1.01^∗^ [1.00, 1.02]	1.00 [0.99, 1.01]	1.01 [1.00, 1.02]
Access to media (no)						
Yes	1.01^∗^ [1.00, 1.01]	0.99 [0.98, 1.00]	1.03^∗^ [1.00, 1.05]	0.99 [0.95, 1.03]	1.03^∗∗∗^ [1.01, 1.04]	1.03^∗∗^ [1.01, 1.04]

Exponentiated coefficients; 95% confidence intervals in brackets. ^∗^*p* < 0.05, ^∗∗^*p* < 0.01, ^∗∗∗^*p* < 0.001.

## Data Availability

Data are available from https://dhsprogram.com/.
